# Predicting functional upstream open reading frames in *Saccharomyces cerevisiae*

**DOI:** 10.1186/1471-2105-10-451

**Published:** 2009-12-30

**Authors:** Selpi, Christopher H Bryant, Graham JL Kemp, Janeli Sarv, Erik Kristiansson, Per Sunnerhagen

**Affiliations:** 1 Department of Applied Mechanics, Chalmers University of Technology, SE-412 96 Göteborg, Sweden; 2 School of Computing, Science and Engineering, University of Salford, Salford, M5 4WT, UK; 3 Department of Computer Science and Engineering, Chalmers University of Technology, SE-412 96 Göteborg, Sweden; 4 Department of Mathematical Sciences, Chalmers University of Technology and the University of Gothenburg, SE-412 96 Göteborg, Sweden; 5 Department of Zoology, University of Gothenburg, Box 463, SE-405 30 Göteborg, Sweden; 6 Department of Cell and Molecular Biology, Lundberg Laboratory, University of Gothenburg, PO BOX 462, SE-405 30 Göteborg, Sweden

## Abstract

**Background:**

Some upstream open reading frames (uORFs) regulate gene expression (i.e., they are functional) and can play key roles in keeping organisms healthy. However, how uORFs are involved in gene regulation is not yet fully understood. In order to get a complete view of how uORFs are involved in gene regulation, it is expected that a large number of experimentally verified functional uORFs are needed. Unfortunately, wet-experiments to verify that uORFs are functional are expensive.

**Results:**

In this paper, a new computational approach to predicting functional uORFs in the yeast *Saccharomyces cerevisiae *is presented. Our approach is based on inductive logic programming and makes use of a novel combination of knowledge about biological conservation, Gene Ontology annotations and genes' responses to different conditions. Our method results in a set of simple and informative hypotheses with an estimated sensitivity of 76%. The hypotheses predict 301 further genes to have 398 novel functional uORFs. Three (*RPC11*, *TPK1*, and *FOL1*) of these 301 genes have been hypothesised, following wet-experiments, by a related study to have functional uORFs. A comparison with another related study suggests that eleven of the predicted functional uORFs from genes *LDB17*, *HEM3*, *CIN8*, *BCK2*, *PMC1*, *FAS1*, *APP1*, *ACC1*, *CKA2*, *SUR1*, and *ATH1 *are strong candidates for wet-lab experimental studies.

**Conclusions:**

Learning based prediction of functional uORFs can be done with a high sensitivity. The predictions made in this study can serve as a list of candidates for subsequent wet-lab verification and might help to elucidate the regulatory roles of uORFs.

## Background

Different genes are expressed differently in different places, at different times and in different amounts. Misregulation of gene expression can cause an abnormality, leading to disease(s) or even cancer [1]. Therefore, a complete understanding of gene regulation is important; one step towards this is to elucidate the roles of post-transcriptional regulatory elements.

**Figure 1:**
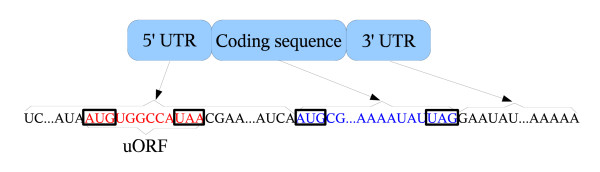
**Schematic representation of mRNA primary structure**. AUG is start codon. A stop codon can be UAA, UAG, or UGA. A 5' UTR may have zero or more uORFs.

Upstream open reading frames (uORFs) are among the post-transcriptional regulatory elements that may be present in the 5' untranslated region (UTR) of mRNA [2] (Figure 1). A 5' UTR region is the region between transcription start site and the main coding sequence (CDS). A uORF is identified by the presence of both a start codon before (i.e., *upstream of*) the start codon of the CDS, and an in-frame stop codon. Research has revealed that the frequency of transcribed uORFs is higher in genes with critical roles, such as homeobox (development-controlling) genes, proto-oncogenes (whose mutation or over-expression can lead to cancer), growth factors, and transcription factors [3]. Furthermore, it has been shown that some transcribed uORFs regulate the translation process (i.e., the uORFs are *functional*) [4-8], while a few others do not (i.e., the uORFs are *non-functional*) [9,10].

Functional uORFs have been shown to play important roles in keeping organisms healthy, usually by controlling the synthesis of certain proteins which are harmful if over-synthesised [11,12]. One example of this is the condition *thrombocythaemia *[[13], accessed on 12 September 2007] where blood contains too many platelets, a type of blood cell involved in blood clotting. People with this condition have a higher risk of developing a blood clot, a stroke or heart attack. The production of platelets is controlled by the hormone expressed from the gene thrombopoietin. According to a review [14], based on [15], under normal conditions, the uORFs of thrombopoietin mRNA act to limit the translation of the thrombopoietin gene and thus limit the production of the platelets in the blood cells. When uORFs are somehow eliminated from the thrombopoietin mRNA, the translation of thrombopoietin gene is increased and thus the amount of the platelets, causing thrombocythaemia.

To date, transcribed uORFs have only been verified in a small number of genes in several organisms. From these data, a partial understanding of how uORFs can regulate protein expression has been achieved. However, as more and more uORFs have been found in the mRNA of genes with critical roles, it has become important to get a complete understanding of how uORFs are involved in gene regulation. To be able to draw a complete understanding of the mechanism, we expect that a large number of experimentally verified functional uORFs will be needed.

Until recently, studies on uORFs have been largely limited to lab-based experiments. The most direct test to verify that uORFs are transcribed and whether they are functional is by comparing the amount of mRNA and the amount of protein produced from the main gene in its proper chromosomal context with and without site-specific mutation(s) on the uORF(s) of interest. The site-specific mutation is usually done on one of the bases of a uORF's start codon to remove the uORF. In general, these experiments to verify that uORFs are transcribed and whether they are functional, are costly and time-consuming (≈ 4 man-months per gene). As a result, the simplest approach to searching for functional uORFs, i.e., by sampling genes at random and testing their uORFs in the laboratory, is not effective, even for the simplest eukaryotic (the yeast *Saccharomyces cerevisiae*) genome. It has been suggested that no more than 10% of the 6000 yeast genes will have one or more functional uORFs and each of these genes will on average have two functional uORFs. Thus, if one searched for functional uORFs by selecting genes at random and testing them in the lab, then on average it would take ≈ 20 man-months to find a single functional uORF. Therefore, an *in silico *prediction method which can help in selecting sets of candidate functional uORFs for lab experimental studies is essential.

This study sets out to develop such a method using a machine learning technique and the yeast *Saccharomyces cerevisiae *as the model organism. Given relevant data, the method should automatically generate hypotheses which can then be used to predict novel functional uORFs.

Although a large number of genomic (DNA) sequences are now available, the task of computationally identifying functional uORFs is still very challenging. As explained earlier, a functional uORF is a transcribed uORF which can regulate the translation of the main gene (the associated CDS). Thus, in order to find functional uORF(s), one should ideally look for uORF(s) in the 5' UTR sequences. However, until recently, the start of 5' UTRs were only known for a small number of genes. Therefore, previous genome-wide computation studies trying to identify functional uORFs used intergenic sequences (sequences between two genes) instead [16,17]. Determining the start of 5' UTRs computationally becomes even more difficult due to the fact that some genes have multiple transcription start sites [18]. This situation makes the task of determining which genes contain uORF(s) in their 5'UTRs very challenging, not to mention identifying which of these uORFs are functional.

In this paper, a new approach to predicting functional uORFs is presented. There are three main differences between this work and the work described in [17]. First, we employ a different machine learning system, Aleph [19]. Second, instead of intergenic sequences, we use 5' UTR sequences. Third, in addition to the knowledge from *S. cerevisiae*'s sequences, knowledge derived from sequences of other yeast species, an analysis of expression data sets, and Gene Ontology annotations are also used to form the background knowledge for Aleph. Why we think these heterogeneous data could be useful for this study and how we transform them into a suitable format for Aleph are discussed in the methods section.

## Methods

### Learning method

Among many machine learning techniques, we chose inductive logic programming (ILP) [20] for the following reasons. First, ILP provides a richer representation than other machine learning techniques which are based on an attribute-value representation that cannot concisely represent the relationships between attributes in the uORF domain. For example, the attribute-value representation cannot concisely represent relationships between uORFs and UTRs because a UTR may have an arbitrary number of uORFs. Second, unlike other machine learning techniques, ILP has a beneficial feature in that it is able to bias inference to take into account background (domain) knowledge from domain experts and/or the literature [21,22]. Third, ILP's input (examples and background knowledge) and output (hypotheses/rules) are all represented in predicate logic. This representation can be easily translated into English. Consequently domain experts can help with the selection and integration of potentially helpful knowledge and the final dissemination of discoveries to the wider scientific community. Lastly, ILP has been successfully applied to a diverse range of real-world problems, such as those reviewed in [23-25], and has been shown to have the potential to help in selecting sets of candidate functional uORFs for lab experimental studies [17].

### Knowledge from 5'UTR sequences of *S. cerevisiae*

The 5' UTR sequences were extracted from upstream sequences of protein coding genes of *S. cerevisiae*. The lengths of 5' UTRs were derived from tiling microarray data presented in [26]. The upstream sequences were downloaded from ENSEMBL database at BioMart [[27], accessed on 15 March 2007] and 5' UTRs longer than 1000 bases were excluded. 1000 bases were chosen because 5' UTR lengths in *S. cerevisiae *are mainly distributed below 500 bases, with a small percentage between 500 and 1000 bases, and only very rarely are they above 1000 bases [18,26]. In total, we used 4,938 5' UTR sequences. uORFs, with minimum length 3 codons (including start codon and stop codon), were extracted from the 5' UTR sequences using *getorf *of the EMBOSS package [28]. The result is 3,647 (21+2+3,624) uORFs from 1,493 *S. cerevisiae *genes (Table 1). 18 of these 1,493 genes had previously been studied in detail and are documented to have uORFs transcribed within their mRNAs, as summarised in [4] and [29]. The detailed composition of the data used for our experiments here is summarised in Table 2.

**Table 1:** Detailed composition of uORFs obtained using getorf of the EMBOSS package.

Number of Genes		uORFs		
		
		Functional	Non-functional	Unlabelled
18 studied	9	21	-	-
genes	2	-	2	-
	7	-	-	8

1,475 other genes		-	-	3,616

1,493 genes		21	2	3,624

**Table 2:** Detailed uORF composition from 18 studied genes within the collection obtained using getorf of the EMBOSS package.

Gene Name	Systematic Name	uORFs		
		
		Functional	Non-functional	Unlabelled
*CLN3*	YAL040C	1	-	-
*GCN4*	YEL009C	4	-	-
*HAP4*	YKL109W	2	-	-
*TIF4631*	YGR162W	5	-	-
*YAP1*	YML007W	1	-	-
*YAP2*	YDR423C	2	-	-
*HOL1*	YNR055C	1	-	-
*PET111*	YMR257C	4	-	-
*CPA1*	YOR303W	1	-	-

*SCO1*	YBR037C	-	1	-
*CBS1*	YDL069C	-	1	-

*INO2*	YDR123C	-	-	1
*PPR1*	YLR014C	-	-	1
*URA1*	YKL216W	-	-	1
*LEU4*	YNL104C	-	-	1
*RCK1*	YGL158W	-	-	2
*DCD1*	YHR144C	-	-	1
*SCH9*	YHR205W	-	-	1

18 Genes		21	2	8

Features that can determine the impact of a uORF on gene regulation, as suggested in [4] and [29], were extracted from the 5' UTR sequences. These include distance from the uORF to the start of the CDS in bases, frequency of AU and GC base pairs immediately upstream and downstream of each uORF, the length of the uORF in codons, number of uORFs found in the UTR, and the length of the UTR. The bases in positions -3 and +4 relative to each uORF were also extracted. Through experiments with mammalian sequences, these positions were found to give an optimum context for an AUG to be recognised by ribosome [30,31]. All of this information was represented as ILP background knowledge [see Additional file 1: Table S-1].

Beside listing the instances of the background predicates, background knowledge in ILP can also be represented as rules. Here, some declarative rules, that are potentially useful for helping to identify functional uORFs, were created to examine uORF's and UTR's features and their relationships. These include rules that examine the abundance of AU and GC base pairs immediately upstream and downstream of each uORF, whether the base in position +4 relative to the uORF's start codon is G and whether the base A or G is present at position -3 relative to the uORF's start codon. These rules are given in [Additional file 1: Table S-2].

### Knowledge from sequences of other yeast species

Most functional genomic elements are needed to preserve fitness and thus are conserved between closely related species. This insight may also apply to functional uORFs, that is to say, uORFs which are functional are likely to be conserved in closely related species; this has been demonstrated by the uORFs of *GCN4 *and *CPA1 *which are conserved in multiple fungal species [[32], Supplementary Material 1], [[16], Figure 1]. Therefore, information about uORFs in closely related species to *S. cerevisiae *could be beneficial for this study. *Saccharomyces *phylogeny is given in [Additional file 1: Figure S-1].

5' UTR sequences of several other species in the genus *Saccharomyces *would be ideal sources, but these are not available. However, upstream sequences of six other *Saccharomyces *species i.e., *S. bayanus, S. castellii, S. kluyveri, S. kudriavzevii, S. mikatae and S. paradoxus *are available. *S. castellii *and *S. kluyveri *are considered quite far from *S. cerevisiae *[33], and thus would be expected to have lesser degree of conservation to *S. cerevisiae*. Of the four closer to *S. cerevisiae, S. paradoxus, S. mikatae *and *S. bayanus *were studied recently in [34]. It was found that the order of genes in these three genomes and *S. cerevisiae *is well conserved. It was also suggested that the three genomes and *S. cerevisiae *have diverged enough to allow functional elements to be recognised. Therefore, we chose to use these three species.

For each *S. cerevisiae *gene, the upstream sequences of 500 bases of the orthologs from *S. paradoxus *[[35], accessed on 3 May 2007], *S. mikatae *[[36], accessed on 3 May 2007] and *S. bayanus *[[37], accessed on 2 May 2007] were downloaded from Saccharomyces Genome Database (SGD) [38]. 500 bases was chosen because 5' UTR lengths in *S. cerevisiae *are mainly distributed below 500 bases [18,26]. Furthermore, according to [33], the mean length of intergenic regions in the *Saccharomyces *family is around 500 bases. From these upstream sequences, uORFs with minimum length 3 codons as well as their features were extracted. This information, which is similar to that of *S. cerevisiae*, was represented as background knowledge [see Additional file 1: Table S-3].

### Conservation testing

In determining whether a uORF is conserved in ortholog genes from different species, we looked for the presence of uORFs of approximately the same length (i.e., the difference in the lengths is not more than three codons; this criterion was used in [32] and [16]) at the same position in the sequence of uORFs relative to the CDS. This was done in preference to using a conventional sequence comparison approach that considers nucleotide sequences in a base-by-base manner. Our new method of testing for conservation is particularly useful for finding regulatory motifs in distantly related species where sequence similarity between the species can be low but the presence of regulatory motifs remains. We implemented this by defining several rules [see Additional file 1: Table S-4].

### Knowledge from Gene Ontology annotations

To facilitate the application of knowledge about one organism when reasoning about another, there has been community effort to create common vocabularies, the Gene Ontology (GO) [39], for describing gene and gene product attributes in any organism. The idea of using GO annotations here is to allow ILP to examine uORFs associated with genes which share the same or related annotation(s). The basis for this is that one may wonder whether uORFs tend to be functional in the UTRs of genes whose products are involved in a specific function(s), or in a specific process(es), or expressed in a specific cellular component(s). As of 12 April 2007, GO contains a total of 22,968 terms (13,464 for biological process, 7,657 for molecular function and 1,937 for cellular component). The terms are arranged in a directed acyclic graph according to the GO hierarchies, i.e., process, function and component ontologies. The three ontologies, were downloaded from GO website [[40], version 26:03:2007]. The ontology for category molecular function consists of 15 levels, for biological process 18 levels and for cellular component 16 levels. The number of nodes in the first five levels in each ontology are shown in Table 3.

**Table 3:** Number of nodes in the first five levels in each of the GO categories.

	Level				
	
GO category	1	2	3	4	5
Molecular Function	1	20	730	737	1531
Biological Process	1	20	677	1892	6149
Cellular Component	1	17	279	902	2125

In this work, we used the GO annotations for yeast genes [[41], version 24 March 2007] provided by SGD [42]. Although not all of the GO terms are used for annotating yeast genes, the GO annotations for yeast can be very specific. There are some annotations that only cover one gene. For the purpose of our study, terms as specific as this are not useful. We want more general annotations for yeast genes, so that each used annotation covers more genes. GO slim [[43], accessed on 11 April 2007] provides such mapping. However, this mapping is too general for our study. Therefore, for each of the GO categories, we mapped the yeast genes to the third level terms of their GO annotations (if the original level of GO annotation is fourth, fifth and so on). The new mapping was given to ILP as background knowledge [see Additional file 1: Table S-5]. Several rules [see Additional file 1: Table S-6] were defined to allow ILP to relate a uORF with one or more function/process/component-annotation(s) of the main gene associated to that uORF.

### Knowledge from expression data

Microarray data can be viewed as a gene expression matrix where each row represents a gene and each column represents a condition, and the value of each position in the matrix represents the expression of a certain gene in a certain condition. Such data allow us not only to study the expression of individual genes under different conditions in a genome-scale, but also allow us to group genes which respond similarly to a set of conditions. With regard to predicting functional uORFs, it has been suggested that the polysomal association study integrating microarray data sets from several different stress conditions can provide an efficient way to experimentally verify the predictions of functional uORFs [16].

Here, derived knowledge from an analysis of four microarray data sets measuring translational activity under rapamycin stress [44], oxidative (*H*_2_*O*_2_) stress [45], butanol stress and amino acid starvation [46] were included as background knowledge for ILP. This was done to investigate whether functional uORFs could be explained in terms of how genes respond to different stress conditions. The polysome-to-monosome log-fold change between stressed and normal condition was used to determine whether the expression of a gene is up/down/not-regulated under each stress. The latter information was given to ILP as background knowledge [see Additional file 1: Table S-7]. We also defined two rules [see Additional file 1: Table S-8] to relate a uORF with information on whether the main gene associated to that uORF is regulated (either up or down) or not regulated under certain stress. A more detailed description of how the microarray data were analysed is given in [Additional file 1: Part III].

## Results

### Leave-one-out cross-validation

Since our goal is to learn how to recognise which uORFs regulate gene expression, we can consider this learning task to be a classification problem. Ideally, a typical classification system in ILP (or machine learning in general) learns from a mixture of positive and negative examples. In this domain, positive examples would be uORFs that are transcribed and regulate gene expression (i.e., functional) and negative examples would be uORFs that are transcribed but do not regulate gene expression (i.e., non-functional). The uORF data from 1,475 genes (Table 1) are all unlabelled. Hence, for the training stage in this study, only the uORF data of the 18 studied genes were used.

As summarised in Table 2, among the uORF data of the 18 studied genes, 21 uORFs have been verified experimentally as functional [4]. These were used as positive examples. [[29], p. 32] pointed out that there are only two uORFs from two genes which have been verified to be non-functional and the 5' UTR of each of these genes does not contain any other uORFs known to be functional. Therefore, there were only two negative examples in our data set. Given the characteristics of the data, we explored the positive-only setting [47] of an ILP system Aleph [19]. The positive-only setting of Aleph allows induction of hypotheses in the absence of negative examples.

We investigated whether our new approach could generate hypotheses with good performance. Only the positive examples of the 18 studied genes were used. Aleph's parameter settings and the definition of hypotheses space are given in [Additional file 1: Tables S-9 and S-10]. Since there were only 21 positive examples, evaluation was done using leave-one-out cross-validation. This means that each example in turn is used as a test set, while the other 20 examples are used as a training set. Thus in total, we did 21 executions.

Since we only used positive examples, the performance of the hypotheses was measured using sensitivity (or recall), which measures the fraction of positives which are recognised by the hypotheses as positives. In 16 executions, from a total of 21, the hypotheses can correctly recognise the test set. Thus, the estimate of how well our hypotheses can correctly identify functional uORFs is 76%.

### Predicting novel functional uORFs

Having achieved reasonably high sensitivity from the experiment detailed in the previous subsection, we conducted a further experiment in which the same background knowledge and ILP settings were used, but this time all of the 21 positive examples were used to generate a set of hypotheses. The English translations of the hypotheses are shown in Table 4.

**Table 4:** The English translations of the hypotheses generated from the set of 21 positive examples.

A uORF has functional role if it satisfies at least one of the following rules.	
1.	the main gene is regulated under butanol stress and the product of the main gene is involved in nucleic acid binding;
2.	the uORF is conserved in two other species, the main gene is localised in intracellular (or protoplasm), and the UTR length > = 463;
3.	the uORF is conserved in three other species and the main gene is localised in intracellular (or protoplasm);
4.	its length < = 7 and the product of the main gene is involved in nucleic acid binding;
5.	the base in position +4 relative to the uORF's start codon is 'G' and the main gene is involved in regulation of biological process;
6.	its length < = 6, the main gene is localised in intracellular (or protoplasm), and the main gene is involved in regulation of biological process;
7.	the uORF is conserved in two other species, the main gene is not regulated under low concentration of H_2_O_2_, the main gene is localised in intracellular (or protoplasm), and the UTR length > = 244;
8.	the product of the main gene is involved in translation regulator activity.

The fact that negative examples were not used during training raises a suspicion that the hypotheses that have been generated could have been overly general. Furthermore, the performance measure used (sensitivity) does not penalise over-generalisation and so will not indicate if over-generalisation has arisen. Note that a hypothesis which simply states that any uORF is a functional uORF would have a sensitivity of 100%. Hence there was a need for an additional test to determine whether this set of hypotheses were overly general i.e., tend to clasify any example as functional uORF.

The uORF data on these 18 studied genes is precious due to its scarcity and therefore it made sense to utilise every part of the limited data available. The set of hypotheses shown in Table 4 was used to classify the negative (2) and unlabelled (8) examples within the 18 studied genes. Only 2 of 10 examples were classified as positives; one from the negative set and one from the unlabelled set. Thus, we believe that the high sensitivity is not because the hypotheses tend to classify any example as positive.

When the same set of hypotheses was used to clasify 3,616 unlabelled examples from 1,475 genes, they predict 398 uORFs from 301 genes as functional. The 398 predicted functional uORFs are listed in additional file 1: Table S-11. Generally, more precise mapping of transcription start sites in yeast will in some cases help confirm whether these uORFs are real or not, and some of these predicted functional uORFs may turn out to be artifact due to errors in the transcription start site prediction. Thus, extensive lab work would be required to verify whether these 398 uORFs from 301 genes are indeed functional. However, there are other observations that provide support for our predictions (see discussion section).

## Discussion

The novel approach to predicting functional uORFs in the yeast *S. cerevisiae *presented here makes use of knowledge about biological conservation, GO annotations, and genes' response to different stress conditions; while there have been several studies involving machine learning which make use of expression data and/or GO annotations (e.g. [48-51]), such a combination of knowledge has not been explored previously for learning yeast functional uORFs.

To date, there are very few computational studies on uORFs. The most closely related work are the studies by [16] and [32], and these are discussed in detail below. [52] Studied uORFs in the genome of fungal pathogen *Cryptococcus neoformans *with the aim of finding the proportion of uORFs conserved in four strains of *C. neoformans*. Similar to [52][53] looked for conserved uORFs between human and mouse genomes.

As in our work, the overall goal of the study in [32] is to find additional genes that are potentially regulated by uORF(s). Both [32] and [16] inspired us to consider uORF conservation in our work, although the way in which conservation is tested here is different, focusing on the relative positions and lengths of uORFs, rather than sequence similarity. Further to their computational work, [32] investigated seven genes experimentally. Of the five genes (*RPC11, TPK1, FOL1, WSC3*, and *MKK1*) that [32] hypothesised may have functional uORFs, three genes, which have one uORF each, were predicted by our hypotheses to have functional uORFs (Table 5). *WSC3 *was excluded from our data, since its UTR length was predicted to be well over 1000 bases based on [26]. The uORFs of *ECM7 *and *IMD4*, which were found by [32] to have little effect on translation, were predicted as non-functional by our hypotheses.

**Table 5:** Comparison between predictions made by our hypotheses (Table 4) and by [32] for the seven genes that they wet-experimentally tested.

Gene Name	Systematic Name	uORF's Position	uORF's Length	Predicted as functional in	
				
				Z&D	This study
*RPC11*	YDR045C	-60	4	Yes	Yes
*TPK1*	YJL164C	-42	5	Yes	Yes
*FOL1*	YNL256W	-65	4	Yes	Yes
*WSC3*	YOL105C	-50	7	Yes	*a*
*MKK1*	YOR231W	-71	10	Yes	No

*ECM7*	YLR443W	-15	5	No^*b*^	No
*IMD4*	YML056C	-99	14	No^*b*^	No

^*a*^5' UTR length was predicted to be well over 1000 bases and thus this gene is not included in this study.

^*b*^Zhang and Dietrich found these uORFs have little effect on translation; we consider them as non-functional.

The work described here provides some improvements compared to [16], and in the remainder of this section we discuss the differences in the methodology, hypotheses and predictions. Concerning methodology, the computational system used in [16] was an expert system shell with a certainty factor model for representing uncertainty in both the data and the rules/hypotheses. To generate the initial hypotheses, a rule base is constructed manually. The final set of hypotheses was generated after several cycles of running the expert system with the input data, analysing the results, and manually modifying the rule base for better classification. In contrast, in the work described here, the hypotheses are generated automatically by the ILP system. Thus, when applying the method for learning functional uORFs in other organisms, applying the method described here will be more practical than the one described in [16].

In [16], each rule for inferring whether a uORF was likely to affect gene expression was assigned a certainty factor representing the confidence in a consequent of the rule being true if all of the antecedents are true. If a uORF was predicted to be functional using two or more different lines of inference, then the certainty factors associated with these were combined, and the resulting combined certainty factor was used to score the uORF. The highest certainty factor value for any one of a gene's uORFs was used as the score for the gene itself, and those genes with a score above a selected threshold were classified as having a functional uORF. In that work, genes could be ranked according to their scores. In contrast, the ILP approach used in this paper gives a boolean "yes or no" prediction for whether each uORF has a functional role, and there are no scores that can be used for ranking. Thus, unlike in [16], there is no notion of having "strongly predicted" genes in the present work. The fact that one approach produced a ranked list of predictions while the other resulted in an unordered set of predictions makes a direct comparison of their results difficult.

While it is plausible that collectively, the rules listed in Table 4 will have a relation to the probability of uORFs having a functional role, it is uncertain at this point how the specific combinations of criteria that make up the rules relate to a biological context. This is primarily due to the small number of examples underlying each rule. Several of the derived criteria are generally in agreement with previously identified properties of genes with functional uORFs. Thus, two of the rules require the 5' UTR length to be over a certain value (Rules 2 and 7), which is consistent with uORFs generally being placed in genes with long untranslated regions. Rule 5 suggested that the base G in position +4 relative to the uORF's start codon is a favourable context for yeast. Previous research on mammals [30,31] have shown that AUG context has influence on the recognition of AUG by the ribosome. The optimum AUG context for mammalian genes was found to be A or G in position -3 and G in position +4, where the A of uORF's AUG is position +1. A or G in position -3 have also been shown as favourable context in yeast too [54]. A requirement for short uORFs has been marked in two rules (Rules 4 and 6). This is in agreement with the finding in [16] that uORFs conserved in evolution between *Saccharomyces *species are shorter than non-conserved (and presumably non-functional) uORFs. It is also interesting to note that several of the rules (Rules 1, 4, 5, 6, and 8) imply a role in regulation of transcription for the gene product. In [16], among genes with predicted functional uORFs, an overrepresentation with products implicated in transcription was found by analysis of GO terms.

By comparing the hypotheses in Table 4 with those described in [16] further, the following observation was made. Although 50 to 250 nucleotides was considered as the optimal distance between a functional uORF and the CDS in [16], this feature does not appear in the hypotheses in Table 4, indicating that the learning system used here considers this feature to be less important. This is due to the use of 5' UTR sequences; around 88% of the 5' UTR sequences of *S. cerevisiae *used here are not more than 250 nucleotides, whereas the intergenic regions used in [16] are generally much longer than 5' UTRs.

There are differences in the predictions described in [16] and those described here. In [16], 245 additional genes were predicted to have 367 new functional uORFs. Among these, 34 uORFs from 32 genes were strongly predicted to be functional. Among the strongly predicted ones, 24 uORFs from 23 genes lie within the 5'UTRs based on calculation from [26]. When we checked how many of these 24 uORFs from 23 genes were also predicted as functional by the hypotheses in Table 4, we found eleven uORFs from genes *LDB17, HEM3, CIN8, BCK2, PMC1, FAS1, APP1, ACC1, CKA2, SUR1*, and *ATH1 *[see Additional file 1: Table S-12]. This suggests that these eleven genes are strong candidates for lab experimental studies. Moreover, *HEM3 *has been investigated by [32] and has been confirmed to have one real uORF.

## Conclusions

We have taken a new approach to learning functional uORFs in the yeast *S. cerevisiae*. The method, which can help to select sets of candidate functional uORFs for lab experimental studies, uses the positive-only setting of an ILP system called Aleph and makes use of knowledge derived from biological sequences of several different yeast species, an analysis of several publicly available expression data sets, and Gene Ontology annotations; this is the first time such a combination of knowledge has been explored for learning yeast functional uORFs. With only a little adjustment and provided the relevant data are available, our method can be applied to the task of learning functional uORFs in other organisms. The heterogeneous knowledge used here allows Aleph to generate a set of hypotheses with reasonably high sensitivity (76%). While the idea of using conservation for learning functional uORFs is not new, the way in which conservation is tested here is new.

Our hypotheses are simple and informative. They are quite specific yet general enough to cover different types of functional uORFs. The hypotheses provide provisional insights into biological characteristics of functional uORFs. These may include being conserved in at least two other yeast species, the main gene's product being involved in regulation of biological process, translation regulator activity, and in nucleic acid binding, as well as the main gene being regulated or not regulated under certain stress.

When the hypotheses were used to predict novel functional uORFs from a set of unlabelled uORFs within the genome of *S. cerevisiae*, they predict 301 further genes to have 398 novel functional uORFs. Three (*RPC11*, *TPK1*, and *FOL1*) of these 301 genes have been hypothesised, following wet-experiments, to have functional uORFs [32]. Finally, a comparison of our predictions here and those in [16] suggests that a set of eleven predicted functional uORFs from genes *LDB17, HEM3, CIN8, BCK2, PMC1, FAS1, APP1, ACC1, CKA2, SUR1*, and *ATH1 *are strong candidates for lab experimental studies. The predicted functional uORFs have yet to be tested biologically. Positive results are certainly hoped for. However, whatever the biological test results would be, we believe these could be used to improve the computational research on uORFs as well as to advance the current knowledge in biology.

## Authors' contributions

S designed and executed the research, analysed and interpreted data and results, and drafted and revised the manuscript. CHB contributed to the design of the study, particularly on the machine learning evaluation, helped draft the manuscript, and revised the manuscript critically. GJLK contributed to the design of the study from a bioinformatics perspective, particularly the idea to use GO, helped draft some sections of the manuscript, and revised the manuscript critically. JS and EK performed microarray analysis, participated in selecting the sequence data to be used, and revised the manuscript critically. PS contributed to the design of the study from a biological perspective, suggested the use of microarray data, and revised manuscript critically. All authors read and approved the final manuscript.

## Supplementary Material

Additional file 1 **This file consists of four parts.** The first part contains the background knowledge, the parameter settings and the definition of hypotheses space for the ILP system used in this study. The second part contains a figure that shows Saccharomyces phylogeny. The third part gives a short description of microarray data analysis. The last part lists the predicted functional uORFs including eleven that our analysis suggests to be strong candidates for wet-lab experimental studies. Click here for file
