# Supplementary material for: Predicting functional upstream open reading frames in Saccharomyces cerevisiae
Source: BMC Bioinformatics. 2009 Dec 30;10:451. doi: 10.1186/1471-2105-10-451 (PMC2813248; doi:10.1186/1471-2105-10-451)
Supplement: Additional file 1 — This file consists of four parts. The first part contains the background knowledge, the parameter settings and the definition of hypotheses space for the ILP system used in this study. The second part contains a figure that shows Saccharomyces phylogeny. The third part gives a short description of microarray data analysis. The last part lists the predicted functional uORFs including eleven that our analysis suggests to be strong candidates for wet-lab experimental studies. [file 1471-2105-10-451-S1.PDF]

Supplementary Materials to: Predicting functional upstream open reading frames in *Saccharomyces cerevisiae*

Selpi, Christopher H. Bryant, Graham J.L. Kemp,
Janeli Sarv, Erik Kristiansson, and Per Sunnerhagen

Contents

I Background Knowledge for ILP **2**

II Supplementary Figure **11**

III Microarray Data Analysis **13**

IV List of Predicted Functional uORFs **15**

References **26**

Part I

Background Knowledge for ILP

Short Introduction to Prolog Syntax

In Tables S-1 to S-8, Prolog syntax is used to describe background predicates and background rules given to ILP. A short introduction to Prolog syntax is given here to help readers understand these Tables.

A Prolog clause is written in the form

$$A{:}-B_1, \ldots, B_n.$$

where $n \geq 0$, and the clause is read as "A if B_1 and ... and B_n". A is called the *head* of the clause. Altogether, the B_i form the *body* of the clause. When $n > 0$, the clause is called a *rule*, and when the body of the clause is empty ($n = 0$), the clause is called a *fact* (in this case, the '$:-$' sign is omitted). Each of the B_i and A is an *atomic formula*.

An atomic formula is expressed as $p(T_1, \ldots, T_n)$, where p is an n-ary predicate name, and $T_1, \ldots, T_n$ are the arguments to the predicate p. An argument can be a variable, a constant, or an atomic formula. A variable begins with a capital letter or an underscore character, followed by zero or more letters, digits, or underscore characters. A constant is usually a number, a "string" (zero or more characters in between two `'` characters, or a sequence of letters and (optionally) digits and underscores, that begins with a lower case letter). For example,

```
uORF('YEL009C_uORF1', 361, 4).
```

is a fact; `'YEL009C_uORF1'` , `361`, and `4` are all constant values. While

```
gcrich_down_up(UORF):-
    context(UORF,Au,Gc,_), Gc > Au,
    up_context(UORF,A,G,_), G > A.
```

is a rule; `UORF`, `Au`, `Gc`, `_`, `A`, and `G` are all variables.

In Prolog, anything follows the '%' sign in the same line that the sign appears is considered to be a comment.

Table S-1: Background predicates representing knowledge derived from 5′UTR sequences.[a]

`uORF(X,Y,Z)`	represents uORF `X` which starts `Y` nucleotides upstream of the coding sequence, with length `Z` codons.
`utr(X,Y,Z)`	represents UTR `X` which has `Y` uORF(s) within `Z` nucleotides upstream of gene `X` (`Z` is the length of 5′ UTR region).
`has_uORF(X,Y)`	represents relation between UTR `X` and uORF `Y`.
`belongs_to(X,Y)`	represents relation between uORF `X` and UTR `Y`.
`context(W,X,Y,Z)`	states that within the 20 nucleotides downstream of `W`'s stop codon, the frequency of AU is `X` and the frequency of GC is `Y`; and the base in position +4 is `Z`.
`up_context(W,X,Y,Z)`	states that within the 20 nucleotides upstream of `W`'s start codon, the frequency of AU is `X` and frequency of GC is `Y`; and the base in position -3 is `Z`.

[a]For each predicate, a set of Prolog clauses representing specific facts was generated by instantiating the variables (the arguments to the predicate) with values. For example, for the first predicate in this table the following facts:

```
uORF('YEL009C_uORF1', 361, 4).
uORF('YEL009C_uORF2', 293, 3).
uORF('YEL009C_uORF3', 176, 4).
uORF('YEL009C_uORF4', 151, 4).
```

are generated to represent the four uORFs of gene YEL009C(*GCN4*).

Table S-2: Background rules regarding knowledge derived from 5′UTR sequences.

```
% Check binary comparison less than or equal.
lteq(X,Y):-
    not(var(X)), not(var(Y)), number(X), number(Y), X =< Y, !.
lteq(X,X):-
    not(var(X)), number(X).

% Check binary comparison greater than or equal.
gteq(X,Y):-
    not(var(X)), not(var(Y)), number(X), number(Y), X >= Y, !.
gteq(X,X):-
    not(var(X)), number(X).

% Check if both downstream and upstream contexts of a uORF are GC-rich.
gcrich_down_up(UORF):-
    context(UORF,Au,Gc,_), Gc > Au,
    up_context(UORF,A,G,_), G > A.

% Check if downstream context of a uORF is GC-rich and
% upstream context of that uORF is AU-rich.
gcrich_down_aurich_up(UORF):-
    context(UORF,Au,Gc,_),  Gc > Au,
    up_context(UORF,A,G,_), G < A.

% Check if both downstream and upstream contexts of a uORF are AU-rich.
aurich_down_up(UORF):-
    context(UORF,Au,Gc,_), Gc < Au,
    up_context(UORF,A,G,_), G < A.

% Check if downstream context of a uORF is AU-rich and
% upstream context of that uORF is GC-rich.
gcrich_up_aurich_down(UORF):-
    context(UORF,Au,Gc,_), Gc < Au,
    up_context(UORF,A,G,_), G > A.

% Check if base 'A' or 'G' can be found in position -3 relative
% to the uORF's start codon.
has_A_or_G_in_Min3(UORF):-
    up_context(UORF,_,_,'A'), !.
has_A_or_G_in_Min3(UORF):-
    up_context(UORF,_,_,'G').

% Check if base in position +4 relative to the uORF's start codon is 'G'.
has_G_in_Plus4(UORF):-
    context(UORF,_,_,'G').
```

Table S-3: Background predicates representing knowledge derived from sequences of other yeast species.[a]

`spar_uORF(X,Y,Z)`	represents uORF `X` of *S. paradoxus* which starts `Y` nucleotides upstream of the ortholog coding sequence, with length `Z` codons.
`spar_utr(X,Y,Z)`	represents UTR `X` of *S. paradoxus* which has `Y` uORF(s) within `Z` nucleotides upstream of the ortholog coding sequence `X`.
`spar_has_uORF(X,Y)`	represents relation between UTR `X` and uORF `Y` of *S. paradoxus*.
`spar_belongs_to(X,Y)`	represents relation between uORF `X` and UTR `Y` of *S. paradoxus*.

[a]For each predicate, a set of Prolog clauses representing specific facts was generated by instantiating the variables (the arguments to the predicate) with values. The predicates used to represent uORFs information in *S. mikatae* and *S. bayanus* are similar to those of *S. paradoxus*, with `spar` is changed into `smik` or `sbay` respectively.

Table S-4: Prolog rules for checking conservation of a uORF.

```
conserved_in_x_species(UORF,3):-
    conserved_in_spar(UORF), conserved_in_smik(UORF),
    conserved_in_sbay(UORF), !.
conserved_in_x_species(UORF,2):-
    conserved_in_spar(UORF), conserved_in_smik(UORF), !.
conserved_in_x_species(UORF,2):-
    conserved_in_spar(UORF), conserved_in_sbay(UORF), !.
conserved_in_x_species(UORF,2):-
    conserved_in_smik(UORF), conserved_in_sbay(UORF), !.
conserved_in_x_species(UORF,1):-
    conserved_in_spar(UORF), !.
conserved_in_x_species(UORF,1):-
    conserved_in_sbay(UORF), !.
conserved_in_x_species(UORF,1):-
    conserved_in_smik(UORF), !.
conserved_in_x_species(UORF,0).

% ASCII codes: 95=_ ; 117=u ; 79=O ; 82=R ; 70=F
conserved_in_spar(UORF):-
    uORF(UORF,_,Len1), name(UORF,AsciiuORF),
    get_index(AsciiuORF,Index1), belongs_to(UORF,UTR),
    utr(UTR,Total1,_), spar_utr(UTR,Total2,_),
    Index2 is (Total2-Total1)+Index1,
    name(Index2,AsciiIndex2), name(UTR,AsciiUTR),
    append(AsciiUTR,[95,117,79,82,70],A),
    append(A,AsciiIndex2,AsciiUORF2),
    name(UORF2,AsciiUORF2), spar_uORF(UORF2,_,Len2),
    diff(Len1,Len2,X), X =< 3.

get_index(AsciiuORF,Index):-
    get_after_underscore(AsciiuORF,AsciiEnd),
    remove_uORF_word(AsciiEnd,AsciiIndex),
    name(Index,AsciiIndex).

get_after_underscore([95|T],T).
get_after_underscore([_|T],T1):-
    get_after_underscore(T,T1).

remove_uORF_word([70|T],T).
remove_uORF_word([_|T],T1):-
    remove_uORF_word(T,T1).

append([],L,L).
append([H|L1],L2,[H|L3]):-
    append(L1,L2,L3).

diff(Len1,Len2,X):-
    Len1 > Len2,
    X is Len1 - Len2, !.
diff(Len1,Len2,X):-
    Len1 < Len2,
    X is Len2 - Len1, !.
diff(_,_,0).
```

`conserved_in_smik/1` and `conserved_in_sbay/1` are defined similarly to `conserved_in_spar/1`.

Table S-5: Background predicates representing knowledge derived from GO annotations for yeast genes.[a]

`function(Gene,GOID)`	represents gene product of `Gene` is involved in a molecular function coded by `GOID`.
`process(Gene,GOID)`	represents gene product of `Gene` is involved in a biological process coded by `GOID`.
`component(Gene,GOID)`	represents `Gene` is expressed in a cellular component coded by `GOID`.

[a]For each predicate, a set of Prolog clauses representing specific facts was generated by instantiating the variables (the arguments to the predicate) with values.

Table S-6: Background rules regarding yeast association to GO.

```
% The main gene associated to that uORF is localised or expressed in...
mainORF_is_in(UORF,Comp):-
    uORF(UORF,_,_),
    belongs_to(UORF,UTR),
    component(UTR,Comp).

% The product of the main gene associated to that uORF is involved in
% process...
mainORF_involved_in_process(UORF,Process):-
    uORF(UORF,_,_),
    belongs_to(UORF,UTR),
    process(UTR,Process).

% The product of the main gene associated to that uORF is involved in
% function...
mainORF_involved_in_function(UORF,Function):-
    uORF(UORF,_,_),
    belongs_to(UORF,UTR),
    function(UTR,Function).
```

Table S-7: Background predicate representing knowledge derived from expression data sets.[a]

`regulated(X,Y,Z)`	states that under stress `Y` (1 for amino acid starvation, 2 for butanol, 3 for low concentration of H_2O_2, and 5 for rapamycin), gene product of `X` is either up-regulated (if `Z` is 1), down-regulated (if `Z` is -1), or not-regulated (if `Z` is 0).

[a]For each predicate, a set of Prolog clauses representing specific facts was generated by instantiating the variables (the arguments to the predicate) with values.

Table S-8: Background rules regarding expression data.

```
% The main gene associated to this uORF is regulated under...
regulated_under(UORF,Cond):-
    uORF(UORF,_,_),
    belongs_to(UORF,UTR),
    regulated(UTR,Cond,Val),
    Val \= 0.

% The main gene associated to this uORF is not regulated under...
not_regulated_under(UORF,Cond):-
    uORF(UORF,_,_),
    belongs_to(UORF,UTR),
    regulated(UTR,Cond,0).
```

Table S-9: Parameter settings for Aleph.

Name	Value	Meaning
**evalfn**	posonly	Aleph learns from positive examples only.
**i**	5	The maximum depth of new variables.
**clauselength**	15	The maximum number of atoms in an acceptable clause.
**nodes**	100,000	The maximum number of nodes explored during clause searching.
**depth**	1,000	The maximum depth of the stack used when proving before backtracking.

Table S-10: Definition of hypotheses space for Aleph.

```
% modeh describes the target predicate to be used in the head of a hypothesis.
:- modeh(1,has_functional_role(+uORF)).
% modeb describes the predicates to be used in the body of a hypothesis.
:- modeb(1,uORF(+uORF,-distancefromstart,-uORFlength)).
:- modeb(1,belongs_to(+uORF,-utr)).
:- modeb(1,utr(+utr,-numberofuORF,-utrlength)).
:- modeb(1,lteq(+distancefromstart,#int)).  :- modeb(1,lteq(+uORFlength,#int)).
:- modeb(1,+distancefromstart= #int).       :- modeb(1,+uORFlength= #int).
:- modeb(1,gteq(+distancefromstart,#int)).  :- modeb(1,gteq(+uORFlength,#int)).
:- modeb(1,lteq(+numberofuORF,#int)).       :- modeb(1,lteq(+utrlength,#int)).
:- modeb(1,+numberofuORF= #int).            :- modeb(1,+utrlength= #int).
:- modeb(1,gteq(+numberofuORF,#int)).       :- modeb(1,gteq(+utrlength,#int)).
:- modeb(1,has_G_in_Plus4(+uORF)).    :- modeb(1,has_A_or_G_in_Min3(+uORF)).
:- modeb(1,gcrich_down_up(+uORF)).    :- modeb(1,gcrich_down_aurich_up(+uORF)).
:- modeb(1,aurich_down_up(+uORF)).    :- modeb(1,gcrich_up_aurich_down(+uORF)).
:- modeb(1,conserved_in_x_species(+uORF,#int)).
% Recall number * is used as each gene may be associated with many GO IDs.
:- modeb(*,mainORF_is_in(+uORF,#goid)).
:- modeb(*,mainORF_involved_in_function(+uORF,#goid)).
:- modeb(*,mainORF_involved_in_process(+uORF,#goid)).
% Recall number 4 is used as there are only 4 different stress conditions.
:- modeb(4,regulated_under(+uORF,#stressid)).
:- modeb(4,not_regulated_under(+uORF,#stressid)).

:- determination(has_functional_role/1,uORF/3).
:- determination(has_functional_role/1,lteq/2).
:- determination(has_functional_role/1,'='/2).
:- determination(has_functional_role/1,gteq/2).
:- determination(has_functional_role/1,gcrich_down_up/1).
:- determination(has_functional_role/1,aurich_down_up/1).
:- determination(has_functional_role/1,gcrich_down_aurich_up/1).
:- determination(has_functional_role/1,gcrich_up_aurich_down/1).
:- determination(has_functional_role/1,has_G_in_Plus4/1).
:- determination(has_functional_role/1,has_A_or_G_in_Min3/1).
:- determination(has_functional_role/1,belongs_to/2).
:- determination(has_functional_role/1,utr/3).
:- determination(has_functional_role/1,conserved_in_x_species/2).
:- determination(has_functional_role/1,mainORF_is_in/2).
:- determination(has_functional_role/1,mainORF_involved_in_function/2).
:- determination(has_functional_role/1,mainORF_involved_in_process/2).
:- determination(has_functional_role/1,regulated_under/2).
:- determination(has_functional_role/1,not_regulated_under/2).
```

`modeb` has the format: `modeb(RecallNumber,Template)`. `RecallNumber` specifies how many times the `Template` can be called successfully; * means the `Template` can be called successfully up to 100 times. `Template` is n-ary predicates, with $n \geq 1$ and each of the arguments is a *variable type* preceded by either a '+' (indicates that the argument should be an input), '-' (indicates that the argument should be an output), or '#' (indicates that the argument should be a constant).

Part II

Supplementary Figure

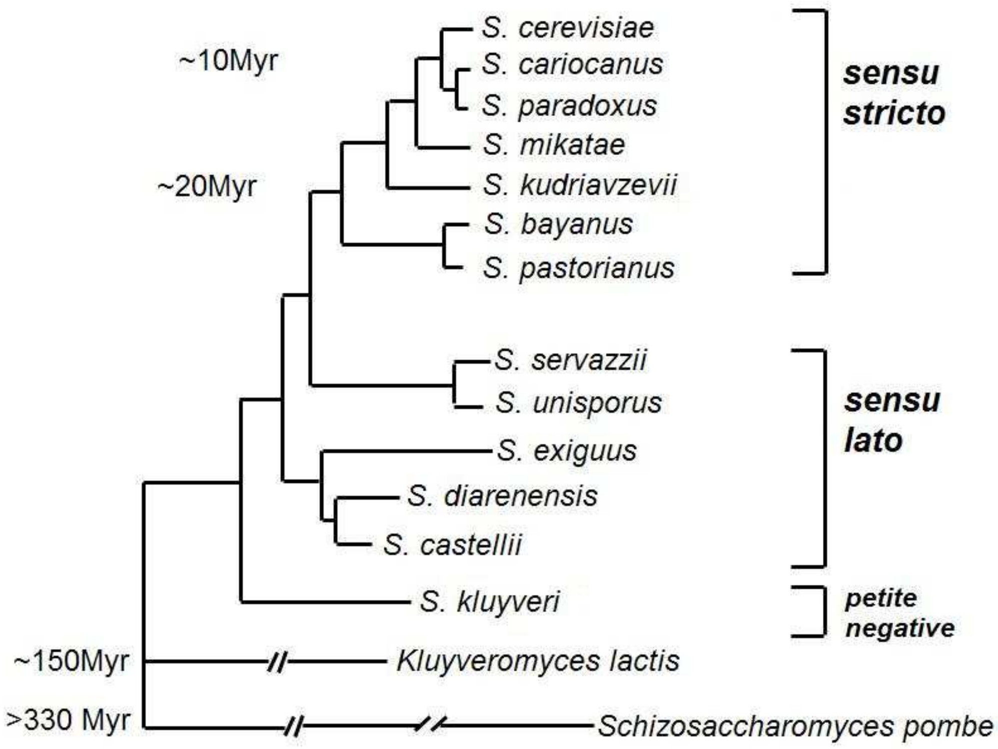

Figure S-1: *Saccharomyces* phylogeny. The figure shows a rough estimation, in million years (Myr), of when evolutionary separation of species took place. (Source: Adapted, with permission, from supplementary materials of [1] shown at `http://www.genetics.wustl.edu/saccharomycesgenomes/yeast_phylogeny.html`).

Part III

Microarray Data Analysis

Short Description of Microarray Data Analysis

Four microarray data sets measuring mRNA levels in polysomal and monosomal fractions between stressed and control conditions were included in the analysis. Publicly available data for oxidative stress [6], butanol stress and amino acid starvation [7] were obtained from ArrayExpress experiment repository [4][1]. Accession numbers for corresponding experiments in ArrayExpress database are E-MEXP-526, E-MEXP-324, E-MEXP-323. Rapamycin data [5] were obtained from Thomas Preiss.

Affymetrix GeneChip expression microarray experiments (i.e., the microarray experiments for amino acid starvation, butanol stress, and oxidative stress) were normalized by using the RMA (Robust Multichip Averaging) method, described in [3]. Loess normalization [8] was applied to two-color cDNA microarray experiments (i.e., the microarray experiments for rapamycin stress).

Polysome-to-monosome (PMF) log-fold changes between stressed and normal conditions were used as measures of translational activity (analogous to "translational state" in [6]).

$$PMF = log\left(\frac{PS/MS}{PC/MC}\right)$$

PMF was defined as the log-ratio of mRNA in polysomal (P) and monosomal (M) fractions between stressed (S) and control (C) conditions. Genes with absolute PMF values above 0.6 were selected for subsequent analysis.

[1] www.ebi.ac.uk/arrayexpress

Part IV

List of Predicted Functional uORFs

Table S-11: 398 Predicted Functional uORFs from 301 Genes.

Gene Name	uORF ID	Distance to CDS (in nucleotides)	uORF's Length (in codons)
YAL015C	uORF10	244	15
YAL047C	uORF2	93	6
YBL034C	uORF3	204	4
YBL064C	uORF1	34	3
YBL088C	uORF2	287	4
YBL091C-A	uORF4	110	14
YBL091C-A	uORF5	63	20
YBL092W	uORF1	317	4
YBR020W	uORF2	140	10
YBR020W	uORF3	71	3
YBR119W	uORF1	151	48
YBR136W	uORF1	139	8
YBR136W	uORF3	64	5
YBR158W	uORF1	216	3
YBR175W	uORF1	8	3
YBR274W	uORF1	19	7
YBR297W	uORF1	11	3
YCL039W	uORF3	85	4
YCR018C	uORF4	220	10
YDL028C	uORF1	17	6
YDL056W	uORF1	170	4
YDL056W	uORF2	162	28
YDL061C	uORF3	376	15
YDL076C	uORF1	103	5
YDL098C	uORF6	91	4
YDL115C	uORF3	329	16
YDL115C	uORF4	251	6
YDL115C	uORF6	32	11
YDL146W	uORF7	52	4
YDL149W	uORF2	236	11
YDL197C	uORF1	443	6
YDL197C	uORF3	410	3
YDL197C	uORF6	187	38
YDL197C	uORF7	168	8
YDL203C	uORF1	99	3
YDL204W	uORF2	465	6
YDL205C	uORF2	129	9
YDL208W	uORF1	44	14
YDR005C	uORF1	121	5

Continued on Next Page...

Table S-11 – Continued

Gene Name	uORF ID	Distance to CDS	uORF's Length
YDR016C	uORF2	8	3
YDR043C	uORF1	172	3
YDR045C	uORF1	60	4
YDR047W	uORF4	144	8
YDR065W	uORF1	17	4
YDR066C	uORF5	590	16
YDR069C	uORF1	11	4
YDR076W	uORF1	8	3
YDR082W	uORF2	13	5
YDR084C	uORF1	7	3
YDR173C	uORF1	43	10
YDR181C	uORF1	14	5
YDR183W	uORF1	17	6
YDR207C	uORF1	123	5
YDR207C	uORF2	17	6
YDR253C	uORF1	118	9
YDR253C	uORF2	105	6
YDR253C	uORF3	77	11
YDR253C	uORF4	70	6
YDR254W	uORF1	100	5
YDR255C	uORF1	30	4
YDR259C	uORF1	663	3
YDR259C	uORF4	483	5
YDR259C	uORF6	168	15
YDR275W	uORF7	293	17
YDR283C	uORF1	8	3
YDR306C	uORF1	48	8
YDR321W	uORF1	104	8
YDR332W	uORF2	16	6
YDR334W	uORF1	52	18
YDR350C	uORF1	14	5
YDR356W	uORF1	267	6
YDR356W	uORF2	205	6
YDR356W	uORF3	88	16
YDR357C	uORF1	66	10
YDR364C	uORF1	23	8
YDR369C	uORF2	82	6
YDR369C	uORF3	72	17
YDR379W	uORF1	11	4
YDR383C	uORF4	65	17
YDR391C	uORF2	233	12
YDR421W	uORF2	46	5

Continued on Next Page…

Table S-11 – Continued

Gene Name	uORF ID	Distance to CDS	uORF's Length
YDR441C	uORF1	31	11
YDR456W	uORF1	7	3
YDR480W	uORF1	49	5
YEL006W	uORF1	32	11
YEL036C	uORF1	201	4
YEL036C	uORF3	20	7
YEL043W	uORF5	58	5
YEL043W	uORF6	8	3
YEL050C	uORF1	7	3
YEL061C	uORF2	109	5
YEL064C	uORF1	11	4
YER053C	uORF1	97	5
YER089C	uORF2	342	16
YER089C	uORF3	332	17
YER098W	uORF1	7	3
YER102W	uORF6	239	6
YER129W	uORF1	340	5
YER129W	uORF2	116	3
YER131W	uORF2	300	5
YER131W	uORF3	240	18
YER132C	uORF1	106	6
YER159C	uORF2	162	3
YER161C	uORF1	49	7
YER162C	uORF2	182	7
YER162C	uORF3	109	6
YER166W	uORF1	206	6
YER167W	uORF1	244	8
YFL027C	uORF1	32	3
YFR005C	uORF1	44	15
YFR017C	uORF2	74	6
YGL006W	uORF1	143	5
YGL013C	uORF1	135	6
YGL031C	uORF2	451	7
YGL031C	uORF4	397	3
YGL031C	uORF8	249	7
YGL037C	uORF1	86	3
YGL071W	uORF1	472	4
YGL071W	uORF2	82	11
YGL085W	uORF2	10	4
YGL094C	uORF2	14	5
YGL094C	uORF3	7	3
YGL120C	uORF1	68	11

Continued on Next Page...

Table S-11 – Continued

Gene Name	uORF ID	Distance to CDS	uORF's Length
YGL134W	uORF2	24	4
YGL151W	uORF1	16	6
YGL162W	uORF1	94	12
YGL162W	uORF2	13	5
YGL197W	uORF1	623	3
YGL197W	uORF2	525	42
YGL197W	uORF4	368	45
YGL197W	uORF5	225	4
YGL197W	uORF6	108	6
YGL222C	uORF1	269	10
YGL222C	uORF2	225	4
YGL222C	uORF3	159	12
YGL238W	uORF1	106	3
YGL240W	uORF1	294	47
YGR027C	uORF4	227	3
YGR027C	uORF8	171	4
YGR027C	uORF9	42	9
YGR042W	uORF1	10	4
YGR062C	uORF4	8	3
YGR071C	uORF1	26	9
YGR098C	uORF1	273	13
YGR100W	uORF1	494	9
YGR100W	uORF3	348	23
YGR100W	uORF4	254	5
YGR122W	uORF1	131	3
YGR126W	uORF3	166	10
YGR134W	uORF11	297	53
YGR134W	uORF12	197	3
YGR134W	uORF3	768	4
YGR134W	uORF6	533	6
YGR134W	uORF7	494	20
YGR134W	uORF9	311	28
YGR142W	uORF6	232	8
YGR148C	uORF1	397	6
YGR148C	uORF2	376	5
YGR148C	uORF5	52	15
YGR188C	uORF1	53	11
YGR222W	uORF1	172	6
YGR222W	uORF2	101	13
YGR238C	uORF5	291	11
YHL002W	uORF3	85	7
YHL007C	uORF1	164	4

Continued on Next Page...

Table S-11 – Continued

Gene Name	uORF ID	Distance to CDS	uORF's Length
YHR050W	uORF1	7	3
YHR072W-A	uORF1	70	5
YHR076W	uORF1	45	3
YHR079C	uORF1	242	8
YHR105W	uORF5	122	4
YHR109W	uORF1	257	22
YHR109W	uORF4	116	5
YHR109W	uORF5	95	12
YHR117W	uORF5	104	5
YHR120W	uORF1	27	6
YHR127W	uORF1	44	3
YHR148W	uORF1	62	3
YHR171W	uORF3	61	5
YHR204W	uORF2	28	9
YIL004C	uORF1	87	7
YIL017C	uORF1	70	15
YIL038C	uORF1	17	6
YIL045W	uORF1	648	3
YIL045W	uORF2	634	6
YIL056W	uORF1	283	26
YIL071C	uORF2	298	7
YIL112W	uORF1	8	3
YIL118W	uORF1	150	10
YIL122W	uORF1	10	4
YIR021W	uORF1	44	4
YJL030W	uORF1	113	10
YJL030W	uORF2	106	3
YJL036W	uORF1	174	7
YJL065C	uORF1	869	4
YJL065C	uORF2	830	4
YJL076W	uORF1	89	3
YJL088W	uORF1	82	7
YJL098W	uORF1	102	7
YJL100W	uORF4	295	16
YJL103C	uORF2	83	3
YJL106W	uORF2	770	3
YJL106W	uORF3	697	23
YJL106W	uORF8	405	6
YJL115W	uORF1	151	10
YJL133W	uORF1	23	3
YJL140W	uORF2	92	9
YJL164C	uORF1	42	5

Continued on Next Page…

Table S-11 – Continued

Gene Name	uORF ID	Distance to CDS	uORF's Length
YJL209W	uORF1	94	10
YJR007W	uORF1	159	3
YJR014W	uORF1	94	4
YJR022W	uORF1	105	3
YJR053W	uORF1	558	97
YJR053W	uORF2	407	4
YJR053W	uORF5	150	6
YJR055W	uORF1	86	3
YJR068W	uORF1	69	3
YJR089W	uORF1	8	3
YJR112W	uORF2	51	5
YJR134C	uORF10	25	9
YJR134C	uORF6	433	7
YJR134C	uORF7	364	3
YJR144W	uORF1	74	6
YKL006C-A	uORF5	219	6
YKL006C-A	uORF7	177	33
YKL006C-A	uORF8	166	3
YKL052C	uORF1	37	6
YKL174C	uORF1	7	3
YKL182W	uORF1	141	7
YKL190W	uORF1	40	13
YKL203C	uORF1	7	3
YKR021W	uORF1	8	3
YKR026C	uORF1	157	5
YKR026C	uORF3	115	8
YKR038C	uORF1	17	6
YKR087C	uORF1	22	4
YLL027W	uORF1	187	8
YLL043W	uORF1	14	3
YLL055W	uORF2	278	3
YLR017W	uORF2	10	4
YLR052W	uORF1	14	4
YLR115W	uORF1	38	3
YLR115W	uORF2	33	6
YLR117C	uORF1	240	3
YLR117C	uORF2	73	10
YLR127C	uORF1	27	5
YLR134W	uORF5	339	20
YLR134W	uORF7	219	7
YLR224W	uORF1	41	14
YLR227C	uORF1	322	11

Continued on Next Page...

Table S-11 – Continued

Gene Name	uORF ID	Distance to CDS	uORF's Length
YLR233C	uORF1	22	8
YLR233C	uORF2	15	4
YLR242C	uORF1	40	7
YLR253W	uORF1	27	7
YLR263W	uORF3	319	8
YLR263W	uORF6	128	15
YLR271W	uORF1	542	10
YLR327C	uORF3	215	3
YLR328W	uORF1	89	3
YLR345W	uORF1	216	5
YLR345W	uORF2	185	6
YLR353W	uORF1	7	3
YLR361C	uORF2	112	3
YLR369W	uORF1	310	10
YLR381W	uORF3	65	3
YLR381W	uORF4	60	20
YLR384C	uORF1	490	133
YLR384C	uORF2	366	4
YLR384C	uORF3	321	11
YLR384C	uORF8	7	3
YLR401C	uORF1	44	5
YLR430W	uORF1	48	16
YML009C	uORF1	10	4
YML015C	uORF1	41	6
YML043C	uORF2	14	5
YML068W	uORF3	60	3
YML078W	uORF4	208	3
YML098W	uORF1	46	6
YML106W	uORF1	45	8
YMR010W	uORF1	228	10
YMR042W	uORF1	69	18
YMR064W	uORF4	419	29
YMR073C	uORF2	209	27
YMR094W	uORF1	10	4
YMR097C	uORF1	449	4
YMR097C	uORF5	356	4
YMR119W	uORF1	11	4
YMR125W	uORF1	95	28
YMR164C	uORF1	11	4
YMR166C	uORF1	201	3
YMR182C	uORF1	16	6
YMR192W	uORF1	56	6

Continued on Next Page…

Table S-11 – Continued

Gene Name	uORF ID	Distance to CDS	uORF's Length
YMR207C	uORF6	23	6
YMR216C	uORF1	189	5
YMR234W	uORF1	81	5
YMR237W	uORF6	132	6
YNL045W	uORF4	30	3
YNL068C	uORF1	11	4
YNL073W	uORF2	10	4
YNL076W	uORF1	157	3
YNL088W	uORF1	331	15
YNL088W	uORF2	202	18
YNL094W	uORF1	154	4
YNL156C	uORF2	167	56
YNL164C	uORF1	19	7
YNL186W	uORF1	127	5
YNL199C	uORF1	75	12
YNL256W	uORF1	65	4
YNL278W	uORF2	212	10
YNL329C	uORF6	250	13
YNL329C	uORF7	47	16
YNR004W	uORF6	32	11
YNR006W	uORF1	30	5
YNR012W	uORF1	60	6
YNR016C	uORF1	341	4
YOL001W	uORF1	511	13
YOL023W	uORF1	17	6
YOL028C	uORF1	7	3
YOL068C	uORF3	482	4
YOL068C	uORF4	188	5
YOL068C	uORF6	79	3
YOL104C	uORF1	238	7
YOL104C	uORF3	209	3
YOL104C	uORF5	52	10
YOL112W	uORF2	8	3
YOL138C	uORF1	57	10
YOL139C	uORF1	88	5
YOR057W	uORF1	67	4
YOR057W	uORF2	56	6
YOR058C	uORF1	8	3
YOR059C	uORF5	373	14
YOR059C	uORF6	256	11
YOR061W	uORF1	102	6
YOR073W	uORF1	36	6

Continued on Next Page...

Table S-11 – Continued

Gene Name	uORF ID	Distance to CDS	uORF's Length
YOR083W	uORF1	60	9
YOR107W	uORF3	7	3
YOR113W	uORF1	87	7
YOR120W	uORF4	484	6
YOR120W	uORF6	236	9
YOR148C	uORF1	7	2
YOR162C	uORF1	221	3
YOR179C	uORF1	56	5
YOR179C	uORF2	45	9
YOR180C	uORF2	360	18
YOR180C	uORF4	190	16
YOR205C	uORF1	8	3
YOR222W	uORF2	393	5
YOR222W	uORF3	386	10
YOR227W	uORF1	36	3
YOR266W	uORF6	126	4
YOR295W	uORF1	74	5
YOR315W	uORF1	58	4
YOR319W	uORF1	67	3
YOR319W	uORF2	56	7
YOR342C	uORF1	91	12
YOR350C	uORF2	25	9
YPL038W	uORF1	99	13
YPL038W	uORF2	8	3
YPL057C	uORF1	144	6
YPL064C	uORF1	28	7
YPL068C	uORF7	73	4
YPL068C	uORF9	57	4
YPL071C	uORF1	16	6
YPL075W	uORF1	705	3
YPL075W	uORF2	697	12
YPL075W	uORF3	479	5
YPL075W	uORF4	376	7
YPL075W	uORF5	308	4
YPL075W	uORF7	278	6
YPL075W	uORF8	101	7
YPL101W	uORF1	7	3
YPL107W	uORF3	416	4
YPL107W	uORF7	117	12
YPL107W	uORF8	41	14
YPL127C	uORF1	44	15
YPL133C	uORF2	162	5

Continued on Next Page. . .

Table S-11 – Continued

Gene Name	uORF ID	Distance to CDS	uORF's Length
YPL140C	uORF2	113	9
YPL146C	uORF1	19	7
YPL167C	uORF1	10	4
YPL181W	uORF1	138	5
YPL199C	uORF1	38	13
YPL202C	uORF1	101	17
YPL246C	uORF1	75	5
YPL247C	uORF1	347	8
YPL270W	uORF2	23	8
YPR005C	uORF2	341	22
YPR005C	uORF3	334	24
YPR018W	uORF1	49	3
YPR021C	uORF2	41	8
YPR026W	uORF1	64	5
YPR034W	uORF1	83	10
YPR042C	uORF1	136	14
YPR057W	uORF1	434	5
YPR057W	uORF2	408	4
YPR061C	uORF5	332	6
YPR068C	uORF1	185	6
YPR152C	uORF1	43	15
YPR173C	uORF3	11	4
YPR193C	uORF8	34	11

Table S-12: Eleven uORFs from Table S-11 that were strongly predicted to be functional in [2]. uORF's length includes start and stop codons.

Gene Name	Systematic Name	uORF ID	Distance to CDS (in nucleotides)	uORF's Length (in codons)
LDB17	YDL146W	uORF7	52	4
HEM3	YDL205C	uORF2	129	9
CIN8	YEL061C	uORF2	109	5
BCK2	YER167W	uORF1	244	8
PMC1	YGL006W	uORF1	143	5
FAS1	YKL182W	uORF1	141	7
APP1	YNL094W	uORF1	154	4
ACC1	YNR016C	uORF1	341	4
CKA2	YOR061W	uORF1	102	6
SUR1	YPL057C	uORF1	144	6
ATH1	YPR026W	uORF1	64	5

References

[1] Paul Cliften, Priya Sudarsanam, Ashwin Desikan, Lucinda Fulton, Bob Fulton, John Majors, Robert Waterson, Barak A. Cohen, and Mark Johnston. Finding Functional Features in *Saccharomyces* Genomes by Phylogenetic footprinting. *Science*, 301:71–76, July 2003.

[2] Marija Cvijovic, Daniel Dalevi, Elizabeth Bilsland, Graham J. L. Kemp, and Per Sunnerhagen. Identification of putative regulatory upstream ORFs in the yeast genome using heuristics and evolutionary conservation. *BMC Bioinformatics*, 8:295, 2007.

[3] R.A. Irizarry, B. Hobbs, F. Collin, Y.D. Beazer-Barclay, K.J. Antonellis, U. Scherf, and T.P. Speed. Exploration, normalization, and summaries of high density oligonucleotide array probe level data. *Biostatistics*, 4(2):249–264, 2003.

[4] H. Parkinson, M. Kapushesky, N. Kolesnikov, G. Rustici, M. Shojatalab, N. Abeygunawardena, H. Berube, M. Dylag, I. Emam, A. Farne, et al. ArrayExpress update–from an archive of functional genomics experiments to the atlas of gene expression. *Nucleic Acids Research*, 2008.

[5] Thomas Preiss, Julie Baron-Benhamou, Wilhelm Ansorge, and Matthias W. Hentze. Homodirectional changes in transcriptome composition and mRNA translation induced by rapamycin and heat shock. *Nat Struct Biol*, 10(12):1039–1047, 2003.

[6] Daniel Shenton, Julia B. Smirnova, Julian N. Selley, Kathleen Carroll, Simon J. Hubbard, Graham D. Pavitt, Mark P. Ashe, and Chris M. Grant. Global Translational Responses to Oxidative Stress Impact upon Multiple Levels of Protein Synthesis. *J Biol Chem*, 281(39):29011–29021, September 2006.

[7] Julia B. Smirnova, Julian N. Selley, Fatima Sanchez-Cabo, Kathleen Carroll, A. Alan Eddy, John E. G. McCarthy, Simon J. Hubbard, Graham D. Pavitt, Chris M. Grant, and Mark P. Ashe. Global Gene Expression Profiling Reveals Widespread yet Distinctive Translational Responses to Different Eukaryotic Translation Initiation Factor 2B-Targeting Stress Pathways. *Mol Cell Biol*, 25(21):9340–9349, November 2005.

[8] G.K. Smyth and T. Speed. Normalization of cDNA microarray data. *Methods*, 31(4):265–273, 2003.
